# Pathogens in Vasculitis: Is It Really Idiopathic?

**DOI:** 10.31662/jmaj.2021-0021

**Published:** 2021-07-09

**Authors:** Chie Miyabe, Yoshishige Miyabe, Ryujin Miyata, Naoko Ishiguro

**Affiliations:** 1 Department of Dermatology, Tokyo Women’s Medical University, Tokyo, Japan; 2 Department of Cell Biology, Institute for Advanced Medical Sciences, Nippon Medical School, Tokyo, Japan

**Keywords:** vasculitis, infection, pathogen, animal models

## Abstract

Vasculitis is an autoimmune disease characterized by the infiltration of leukocytes in blood vessels.

An increasing number of studies on human and animal models have implicated various microorganisms in the pathogenesis of vasculitis. Previous studies have shown the presence of infectious agents, including viruses, bacteria, and fungi, in diseased vessels. However, despite continued research, the link between infection and vasculitis is not fully understood, possibly owing to the lack of appropriate animal models that mirror human disease and the technical limitations of pathogen detection in blood vessels. Among the pathogen-induced animal models, *Candida albicans* water-soluble fraction (CAWS)-induced coronary arteritis is currently considered one of the representative models of Kawasaki (KD) disease. Advances in metagenomic next-generation sequencing have enabled the detection of all nucleic acids in tissue, which can help identify candidate pathogens, including previously unidentified viruses. In this review, we discuss the findings from reports on pathogen-associated vasculitis in animal models and humans, with a specific focus on the investigation of the pathogenesis of vasculitis. Further studies on animal models and microbes in diseased vessels may provide important insights into the pathogenesis of vasculitis, which is often considered an idiopathic disease.

## 1. Introduction

Vasculitis is an autoimmune disorder characterized by the presence of inflammatory leukocytes in blood vessels with destructive damage caused to the mural structures. The nomenclature and definition of vasculitis were first proposed at the International Chapel Hill Consensus Conference held in 1994 and updated in 2012 to incorporate advances based on recent developments in medical expertise ^(1)^. Vasculitis is categorized by size, type, and location according to the type of affected vessels: (i) small vessels, e.g., ANCA-associated and immunoglobulin A (IgA) vasculitis; (ii) medium vessels, e.g., polyarteritis nodosa (PAN) and Kawasaki disease (KD); and (iii) large vessels, e.g., Takayasu arteritis (TA) and giant cell arteritis (GCA) ^(1)^.

The etiology of vasculitis remains poorly understood, and multiple vasculitis-related syndromes are considered idiopathic. For instance, it is estimated that 45%-55% of cutaneous vasculitis cases is idiopathic, 15%-20% is caused by infections, 15%-20% is secondary to inflammatory diseases, 10%-15% is drug-induced, and less than 5% is associated with malignancy ^(2)^. However, an increasing number of human and animal studies suggest that vasculitis is not only caused by a few infectious agents but also, in fact, associated with various pathogenic agents, including viruses, bacteria, fungi, and parasites. The coronavirus disease 2019 (COVID-19) epidemic has attracted an unanticipated interest in infection-associated inflammatory autoimmune diseases, including vasculitis. During the pandemic, children were reported to develop vasculitis-like inflammatory symptoms with clinical features similar to those of KD. This condition was described as multisystem inflammatory syndrome in children (MIS-C) and has been associated with severe acute respiratory syndrome coronavirus 2 (SARS-CoV-2) exposure ^(3)^. Comparable to other autoimmune conditions, an aberrantly activated host immune response to the pathogen is considered to play a central role in vasculitis.

In this review, we summarize previous reports on animal vasculitis models and human vasculitis cases associated with infectious agents and discuss the potential approach for investigating the pathogenesis of vasculitis, which is often considered an idiopathic disease.

## 2. Animal Models of Pathogen-Associated Vasculitis

Animal models are useful tools for improving our understanding of the pathways and molecules that might be involved in disease induction. Compared with models of other autoimmune disorders, such as multiple sclerosis and rheumatoid arthritis, the animal models of vasculitis are less frequently studied. Notably, among the multiple experimental models of vasculitis that have been developed, several are induced by pathogens, including viruses, bacteria, and fungi (Table 1). For example, the injection of a *Candida albicans* water-soluble fraction (CAWS) in mice led to the inflammation of the aortic root and coronary arteries. This can be used as an appropriate model for KD ^(4)^. A deeper understanding of the mechanism by which pathogens induce vasculitis could help develop novel therapeutic targets and prevention strategies for vasculitis. In this section, an overview of previously reported animal models of vasculitis induced by pathogens has been provided.

**Table 1.** Animal Models of Pathogen-Associated Vasculitis.

**Viruses**	PRRSV	Pig	Aorta, renal arteries, postcapillary venules in the dermis
	Aleutian mink disease virus	Mink	Necrotizing arteritis of small muscular arteries
	Equine arteritis virus	Equine	Small-vessel vasculitis throughout the body
	Newcastle disease virus	Chicken	Cerebral vasculitis
	Lymphocytic choriomeningitis virus	Mouse, hamster	Glomerulonephritis and widespread vasculitis
	Cytomegalovirus	Mouse	Aortitis and pulmonary arteritis
		
**Bacteria**	*Chlamydia pneumoniae*	Rabbit	Aortitis
	*Borrelia burgdorferi*	Rat, mouse	Arthritis, myocarditis, and vasculitis (model of Lyme disease)
	BCG + *Mycobacterium intracellulare*	Mouse	Coronary arteritis
	*Mycoplasma gallisepticum*	Turkey	Cerebral arteritis
	*Lactobacillus casei*	Mouse	Coronary arteritis
		
**Fungi**	*Candida albicans*	Mouse	Coronary arteritis
	*Candida krusei*	Mouse	Coronary arteritis

PRRSV, porcine reproductive and respiratory syndrome virus; BCG, Bacillus Calmette-Guérin

### 2.1 Viruses

Numerous animal models of vasculitis associated with viral infection have been studied, mostly in the 1970s-2000s. For instance, porcine reproductive and respiratory syndrome virus (PRRSV) induces systemic necrotizing vasculitis involving the skin and kidneys in pigs ^(5)^. This phenomenon was observed during studies on the porcine disease that spread across Europe and North America in the 1990s.

PRRSV antigens were detected by immunohistochemistry in macrophages surrounding the blood vessels in the skin and kidneys. Aleutian mink disease, caused by the Aleutian disease virus, is a persistent viral infection of minks characterized by prolonged clinical course, progressive weight loss, splenomegaly, lymphadenopathy, hepatomegaly, glomerulonephritis, and necrotizing arteritis ^(6)^. The formation of arterial lesions could be prevented by immunosuppressive therapy. The percentage of minks with arterial disease following experimental infection varied from 19% to 40%. Immunoglobulin G (IgG), complement component 3 (C3), and, occasionally, viral antigens are deposited in the affected glomerular capillaries, suggesting that the deposition of antigen-antibody complexes is the causal factor of arteritis ^(7)^. Equine arteritis virus (EAV), the causative agent of equine viral arteritis, is a small, single-stranded RNA virus related to coronaviruses and toroviruses ^(8)^. EAV-infected horses develop rapid-onset fever, depression, leukopenia, and necrotizing arteritis. Adult horses generally make an uneventful recovery after a viremic phase that may persist for up to 40 days after infection. Chickens infected with the Newcastle disease virus develop pneumonitis followed by severe encephalitis. Histologically, encephalitis is characterized by neuronal degeneration and proliferative vasculitis in the cerebellum, and viral antigens in the neurons, glial cells, and endothelial cells are detected ^(9)^. Mice congenitally or neonatally infected with lymphocytic choriomeningitis virus (LCV) develop gromerulonephritis and necrotizing vasculitis of small- and medium-sized arteries, especially in the kidneys and spleen ^(10)^. Hamsters with persistently high levels of LCV viremia develop chronic glomerulonephritis and widespread vasculitis, whereas hamsters with cleared infection do not develop lesions ^(11)^. Murine cytomegalovirus (MCMV)-infected mice showed a high prevalence of arteritis in both the aorta and pulmonary arteries, with mononuclear cell infiltrates detected in the intima and adventitia ^(12)^. The distribution of the cellular infiltrate affects the adventitial surface more severely. Lymphocytes predominantly infiltrate the adventitia, whereas fewer lymphocytes and more macrophages are detected in the medial and intimal infiltrates. MCMV antigens have also been detected in the walls of affected blood vessels.

### 2.2 Bacteria

*Chlamydia pneumoniae* is a common human respiratory pathogen that has also been associated with atherosclerosis. New Zealand white rabbits intranasally inoculated with *C*.* pneumoniae* showed inflammatory changes in the aorta ^(13)^. Immunohistochemical analysis with an anti-*Chlamydia* antibody yielded positive results in some aortic endothelial cells ^(14)^.

*Borrelia burgdorferi* is primarily responsible for human Lyme borreliosis. LEW/N rats intraperitoneally inoculated with *B*.* burgdorferi* developed Lyme disease-like symptoms, such as arthritis, tendonitis, bursitis, myocarditis, and aortitis ^(15)^. In the early stage of the disease, *B*.* burgdorferi* was detected in the endothelium in the periarticular connective tissues, synovium, and tendons. In the late stage, the titer of IgG antibodies against *Borrelia* antigens increased with time, indicating the continued antigenic stimulation from persistent *Borrelia* infection. Accordingly, systemic inflammation symptoms, including vasculitis, appeared to be secondary to *Borrelia* infection in the tissues and immune response to the same. In the mouse model of Lyme borreliosis (in C3H/He mice), vasculitis of medium and large arteries in cardiac and knee lesions was observed ^(16)^. Mice inoculated with Bacillus Calmette-Guérin followed by booster immunizations with *Mycobacterium intracellulare* developed coronary arteritis ^(17)^. In another study, turkeys were intravenously injected with various doses of *Mycoplasma gallisepticum*, the causative agent of chronic respiratory disease in birds ^(18)^. The arteries of the brain were primarily affected among those in multiple organs, including the heart, liver, lung, spleen, kidney, and gastrointestinal tract. It was demonstrated that viable *Mycoplasma* can induce arteritis. In addition, the protective effect of the lesions was observed by the administration of tetracycline and gold thiomalate. The intraperitoneal injection of nonviable *Lactobacillus casei* cell-wall extract (LCWE) induced coronary arteritis in mice ^(19)^. Arteritis is accompanied by the disruption of the arterial intima and media with the formation of aneurysm, the hallmark of KD in children. This unique mouse model was dependent on the signaling of TLR2 and MyD88 and the subsequent release of pro-inflammatory cytokines, including IL-1β, IL-6, and TNF ^(20)^.

### 2.3 Fungi

Over the last few decades, several murine models of KD have been developed, including the CAWS- and LCWE-induced vasculitis models, which share pathological features with the human disease. Previous studies have suggested that exposure to wind-borne *Candida* might trigger KD ^(21)^. CAWS is a mannoprotein-β-glucan complex secreted by *C*.* albicans* that induces cardiac arteritis resembling the symptoms of human KD when intraperitoneally injected into mice. CAWS is noninfectious since it does not contain live fungal cells, and it is considered a pathogen-associated molecular pattern (PAMP) that activates the innate immune response. CAWS-induced coronary arteritis is widely used and considered an appropriate model for studies on the pathogenesis of arteritis and for developing novel treatments ^(22), (23)^. We have previously shown that the dectin-2-mediated induction of CCL2 production by resident macrophages initiates vascular inflammation followed by IL-1β secretion in a dectin-2/Syk/NLRP3 inflammasome-dependent pathway ^(4)^.

## 3. Pathogen-Associated Vasculitis in Humans

Various pathogens are reported to induce vasculitis in humans. Viruses are primarily associated with small-vessel vasculitis, whereas bacterial infections affect vessels of all sizes, including the aorta ^(24)^. Fungal infection is usually associated with large-vessel vasculitis. In this section, we classify pathogen-associated vasculitis in humans based on the type of microorganism (Table 2), including SARS-CoV-2, reported in previous and recent studies.

**Table 2.** Human Vasculitis Associated with Pathogen.

**Viruses**	HBV	PAN, cryoglobulinemic vasculitis
	HCV	Cryoglobulinemic vasculitis
	HIV	Large-, medium-, and/or small-sized vessel vasculitis, cerebral vasculitis, cryoglobulinemic vasculitis
	VZV	Small- and large-vessel vasculitis of the cerebrum, retina, choroid, kidneys, and skin
	Cytomegalovirus	Vasculitis of the gastrointestinal tract, central nervous system, retina, and cutaneous tissue
	HTLV-1	Necrotizing retinitis, cutaneous vasculitis
	EBV	Leukocytoclastic vasculitis, granulomatous vasculitis, large-vessel vasculitis
	Parvovirus B19	IgA vasculitis, PAN, Kawasaki disease, Wegener’s granulomatosis, GCA, cryoglobulinemic vasculitis
	Hantavirus	Cutaneous vasculitis
	Herpes simplex virus	Necrotizing vasculitis of small- and medium-sized lung and peripancreatic arteries
	Rubella virus	Cutaneous vasculitis
	Coronavirus	Kawasaki disease, MIS-C
		
**Bacteria**	*Staphylococcus aureus*	Aortitis, GPA, Kawasaki disease
	*Streptococcus species*	IgA vasculitis, PAN, KD
	* Bartonella henselae*	Small-vessel vasculitis, endocarditis
	*Mycobacterium tuberculosis*	Takayasu arteritis; IgA vasculitis; cerebral, cutaneous, and retinal vasculitis
	*Salmonella*	Aortitis
	*Clostridium*	Aortitis
	*Burkholderia*	GCA
	*Mycoplasma*	IgA vasculitis, cerebral vasculitis, KD
		
**Fungi**	*Aspergillus*	Cerebral vasculitis, endocarditis
	*Coccidioides*	Cerebral vasculitis, endocarditis
	*Candida species*	Cutaneous small-vessel vasculitis, cerebral vasculitis, endocarditis
		
**Others**	*Treponema pallidum*	Aortitis, retinal vasculitis, cutaneous small-vessel vasculitis
	*Orientia tsutsugamushi*	Systemic vasculitis
	*Borrelia burgdorferi*	Cerebral and retinal vasculitis, GCA

HBV, hepatitis B virus; HCV, hepatitis C virus; HIV, human immunodeficiency virus; VZV, varicella-zoster virus; HTLV-1, human T-cell leukemia virus type 1; EBV, Epstein-Barr virus; PAN, polyarteritis nodosa; GPA, granulomatosis with polyangitis; GCA, giant cell arteritis; MIS-C, multisystem inflammatory syndrome in children

### 3.1 Viruses

The viruses associated with human vasculitis are surprisingly diverse. Hepatitis B virus (HBV) is associated with two types of vasculitis: cryoglobulinemic small-vessel vasculitis and PAN. Immune complex (IC)-mediated small-vessel vasculitis is reported in approximately 10% of patients with HBV infection ^(25)^. The association between PAN and HBV has been frequently reported (10%-54%), usually within the first 6 months of HBV infection ^(26)^. The detection of the hepatitis B surface antigen in the vessel wall has been reported in approximately 30% of patients with systemic vasculitis and in up to 50% of patients with PAN ^(27)^. Clinical HBV-associated PAN is barely distinguishable from classic PAN; however, relapses are considerably rare ^(28)^. Chronic hepatitis C virus (HCV) infection can manifest as mixed cryoglobulinemia (MC), which has been detected in over 50% of patients with HCV infection. Approximately 5% of patients with HCV-associated MC develop cryoglobulinemic vasculitis, owing to circulating IC deposition in small vessels ^(29), (30)^. Several types of vasculitis are associated with HIV infection. Systemic necrotizing vasculitis, leukocytoclastic vasculitis, cryoglobulinemia, central nervous system (CNS) vasculitis, and PAN have been reported ^(31)^. PAN in patients with HIV affects the neuromuscular system and skin more frequently than other organs. HIV antigens and particles are detectable in the vessels in such patients ^(32)^. Virus replication with a direct injury of the vessel wall or IC deposition in the vessel wall is presumed. Varicella-zoster virus has been associated with GCA and vasculitis that affects the CNS, retinal and choroidal small vessels, skin, and kidney ^(25), (33)^. Cytomegalovirus (CMV) infection can lead to vasculitis in various organs associated with the gastrointestinal tract, CNS, retina, and cutaneous tissue ^(25)^. As CMV is detected in 50%-55% of healthy vessels ^(34)^, whether it is the causative agent of vasculitis or simply a less harmful commensal is unclear. Human T-cell lymphotropic virus type 1 (HTLV-1) infection causes adult T-cell leukemia, an aggressive malignancy of CD4-positive lymphocytes. HTLV-1-associated uveitis is a common ophthalmic manifestation of this viral infection; however, retinal vasculitis has also been reported ^(35)^. HTLV-1-associated cutaneous lymphocytic vasculitis, in which malignant T cells infiltrate the skin, has also been reported ^(36)^. Epstein-Barr virus (EBV), which causes various human B-cell lymphomas and infects B cells, has been implicated in the pathogenesis of leukocytoclastic and granulomatous vasculitis, as well as in vasculitis characterized by widespread large-vessel and coronary artery aneurysms ^(37)^. It is also associated with KD ^(38)^. Various autoimmune diseases, including juvenile idiopathic arthritis, systemic lupus erythematosus, systemic sclerosis, reactive arthritis, Sjogren’s syndrome, polymyositis, dermatomyositis, and vasculitis, have been associated with parvovirus B19 infection ^(39)^. The association between parvovirus B19 infection and vasculitis has been reported in Henoch-Schönlein purpura, PAN, KD, Wegener’s granulomatosis, GCA, and cryoglobulinemic vasculitis^(25), (39), (40)^. Parvovirus B19-associated vasculitis is considered to result from direct injuries in the infected vessel wall. Acute hantavirus infection has been associated with cutaneous vasculitis ^(41)^. Necrotizing vasculitis of small- and medium-sized lung and peripancreatic arteries was observed in a neonatal patient with herpes simplex infection ^(42)^. An infant with congenital rubella syndrome showed cutaneous vasculitis ^(43)^.

The association between New Haven coronavirus and KD has been reported in studies since the 1970s ^(44), (45)^. However, these findings received limited attention until the recent COVID-19 outbreak. In the early stages of the COVID-19 outbreak, pediatricians reported MIS-C, which has features similar to those of KD and toxic shock syndrome ^(46)^. Compared to that in severe COVID-19, the polymerase chain reaction (PCR) cycle thresholds for SARS-CoV-2 were higher in MIS-C, indicating a reduced viral burden and validating the concept that MIS-C can occur in response to an infection ^(47)^. Notably, patients with MIS-C not only exhibited appropriate antibody responses to SARS-CoV-2 but also produced autoantibodies specific for endothelial, gastrointestinal, and immune-cell antigens ^(48)^.

Overall, a wide variety of viruses have been implicated in vasculitis by different mechanisms. For example, direct endothelial cell invasion can be undertaken by various viruses, such as HBV, HIV, CMV, and parvovirus B19. IC deposition in the vessel wall and subsequent complement activation and immune-cell recruitment are important processes in cryoglobulinemic vasculitis in HCV-associated vasculitis. In addition, the invasion of malignant CD4-positive lymphocytes infected with HTLV-1 can also lead to vasculitis. However, it should be noted that only a limited number of viruses have been identified using traditional techniques such as enzyme-linked immunosorbent assay, immunohistochemistry, or PCR, which constitute ~0.07% of all viral entities ^(49)^. In other words, at this point, the chances of identifying more unknown pathogenic viruses in the vessels cannot be ruled out.

### 3.2 Bacteria

The incidence of vasculitis induced by bacterial infection is assumed to have decreased from that reported half a century ago. Vasculitis associated with *Staphylococcus aureus* infection is usually a complication resulting from bacterial dissemination and direct invasion of the damaged vessel wall with the formation of a “mycotic aneurysm,” which is most commonly detected in the aorta ^(25)^. In granulomatosis with polyangiitis, the chronic nasal carriage of *S*.* aureus* is linked to disease activity and relapses that can be reduced with antibacterial treatment ^(50)^. Staphylococcal infection triggers the release of proteinase 3 and reactive oxygen species from neutrophils, and superantigens, such as staphylococcal protein A, trigger the activation of auto-specific T and B cells ^(51)^. The metagenomic analysis of intestinal microbiota suggested that the number of sequencing reads with similarity to *Streptococcus* species markedly increased during the acute phase in patients with KD ^(52)^. The correlation between *Streptococcus* infection and IgA vasculitis is widely acknowledged ^(53)^. Streptococcal antigens, such as nephritis-associated plasmin receptor and IgA-binding M proteins, have been identified in the kidneys of patients with IgA vasculitis. *Streptococcus* species have also been associated with PAN and KD ^(24), (26)^. *Bartonella henselae* is the primary causative agent of cat scratch disease, which presents with suppurative lymphadenopathy. It has also been associated with glomerulonephritis, small-vessel vasculitis, and endocarditis in immunocompromised patients ^(54)^. TA has been linked to *Mycobacterium tuberculosis* infection, perhaps via cross-reactivity against vascular peptides that mimic the antigens of *M. tuberculosis*. *IS6110* and *HupB* sequences, which are typically used to identify *M. tuberculosis*, were detected in 82% of tissue samples collected from patients with tuberculosis, 70% of samples from patients with TA, and 32% of samples from patients with atherosclerosis ^(55)^. IgA vasculitis ^(56)^ and cerebral ^(57)^, cutaneous ^(58)^, and retinal vasculitis ^(59)^ have also been associated with *M. tuberculosis* infection. *Salmonella* aortitis is a known complication of *Salmonella* infection that typically affects the abdominal aorta and requires surgical intervention ^(60)^. *Clostridium septicum* arteritis is also a rare life-threatening arterial infection often associated with gastrointestinal or hematological malignancy ^(61)^. Koening et al. showed that a *Burkholderia*-like strain is the causative agent of GCA ^(62)^. This was the first report to directly demonstrate the effects of bacterial infection in vasculitis; however, further studies need to confirm the findings. Infection with *M. pneumoniae* has been associated with Henoch-Schönlein purpura (now referred to as IgA vasculitis), CNS vasculopathy, and KD ^(30)^.

### 3.3 Fungi

Although large-vessel vasculitis caused by fungal infection has rarely been reported in recent years, it was previously reported in immunocompromised patients or as a complication in cardiovascular surgery ^(24)^. Most cases involved endocarditis; however, several cases of invasive fungal vasculitis or meningitis, primarily caused by *Aspergillus* and* Coccidioides*, have also been reported ^(63), (64)^. These fungi invade vessels and cause thrombosis and infarction.

In contrast to findings from animal models, the association between *Candida* infection and coronary arteritis has not been confirmed in humans. However, invasive *Candida* infection causes cutaneous leukocytoclastic vasculitis, owing to the invasion of the blood vessels by pseudohyphae ^(65)^.* Candida* endocarditis and meningitis have also been reported ^(66), (67)^.

### 3.4 Other pathogens

Cardiovascular involvement reported later in the course of untreated syphilis, such as aortitis of ascending aorta, has been estimated to occur in 10% of untreated syphilis cases ^(68)^. Retinal and cutaneous small-vessel vasculitis have also been reported ^(69)^. Scrub typhus, a disease caused by mite-borne rickettsia, is an acute febrile disease caused by *Orientia tsutsugamushi*. Since the vascular endothelium is the principal target site of the organism, it is considered to affect nearly every organ system, leading to varied clinical manifestations ^(70)^. Lyme disease, a common tick-borne infection in the northern hemisphere, is caused by *B. burgdorferi*. CNS involvement, primarily represented by neuroborreliosis, is characterized by perivascular and vascular lymphocytic infiltration associated with the presence of *B. burgdorferi* DNA ^(71)^. Retinal vasculitis has also been reported ^(72)^. A case report on GCA, in which spirochetes compatible with *Borrelia* species were identified in temporal artery biopsy specimens and blood culture samples, has also been published ^(73)^.

## 4. Discussion

The COVID-19 pandemic that started in December 2019 notified that the greatest threat to humanity is still infectious diseases in the modern era. Infections can trigger autoimmune diseases, including vasculitis, in genetically susceptible individuals by modulating host immune responses.

Animal models of vasculitis provide valuable information that can help elucidate the mechanism underlying the pathogenesis of vasculitis. These models have also helped identify novel therapeutic targets. Multiple theories have been proposed to explain how infections induce autoimmune diseases; the potential mechanisms include bystander activation, pathogen-induced necroptosis, superantigen cross-linking, and molecular mimicry ^(74)^. In the human and animal models of pathogen-induced vasculitis, although direct infection of endothelial cells by pathogens has been observed in multiple cases, the exact mechanism remains not completely understood. Most animal models, especially of virus- or bacteria-induced vasculitis, were actively studied until the 1990s. However, the models have been less frequently studied in the last two decades. The challenges in handling larger experimental animals and harmful pathogens, inconsistency with the human disease, and potential ethical issues could be some of the reasons for this. Alternatively, animal models of LCWE- or CAWS-induced KD have been extensively studied in the last two decades. A common characteristic of these models is that neither LCWE nor CAWS is inherently infectious and are only the cell-wall extracts of *Lactobacillus casei* and *C. albicans*, respectively*.* Therefore, these agents are considered superantigens or PAMPs. The advantages of these models include high reproducibility and similarity to human KD, owing to which these can be used to assess novel therapeutic interventions for KD. However, an important caveat is that neither of the two bacteria has been identified in human KD, which may occasionally give rise to concerns that the models do not faithfully represent human KD. Ideally, animal models of diseases should mirror the pathogenesis and mechanism of human diseases. Although these animal models are not identical to human vasculitis, they remain useful as long as the differences are known and the results are carefully interpreted. Indeed, studies on the animal models of KD have led to the discovery of the essential role of IL-1β and the development of clinical trials of anakinra for treating children with KD. Continued effort is necessary to develop appropriate animal models of vasculitis that represent human diseases more faithfully and design alternative experimental systems to minimize animal suffering.

Considering infectious entities as potential causative agents of vasculitis is important. As described in this review, numerous microorganisms have been associated with human vasculitis. The clinical features vary according to the types of organisms, and even the same microorganism can cause different types of vasculitis. Tissue culture techniques, pathogen-specific histochemistry, or PCR with species-specific primers are typically employed to detect the causative pathogens ^(34)^. Given that only a limited number of viruses have been detected using traditional techniques, unidentified viruses that can cause vasculitis may exist. Universal approaches involving the genetic sequencing of bacterial DNA, most commonly 16S ribosomal RNA gene sequencing for bacterial identification, have been widely adopted. Despite continued research, most studies have failed to detect the causative pathogens in human vasculitis, and the link between infection and vasculitis remains incompletely understood. For instance, the DNA sequencing of temporal artery biopsy samples from 17 patients with GCA and 5 controls did not reveal any distinctive microbiome signature in the patients ^(75)^. However, the results of this study were influenced by the use of fixed and paraffin-embedded tissues, in which the DNA may be degraded. Moreover, only DNA microbes, such as bacteria, and DNA viruses can be detected using this method. Since vasculitis is occasionally associated with RNA viruses, including HCV, HIV, and even coronaviruses, as described in the present review, metagenomic next-generation sequencing that facilitates the unbiased characterization of all nonhuman nucleic acids could be beneficial for identifying the potential candidate pathogens in a sample. Further studies are necessary to confirm the presence of previously unidentified pathogens in human vasculitis, which is often considered an idiopathic disease. Further research is also necessary to verify the role of the pathogens in animal models to establish novel therapeutic strategies for pathogen-associated vasculitis.

## Article Information

### 

This article is based on the study, which received the Medical Research Encouragement Prize of The Japan Medical Association in 2020.

### Conflicts of Interest

None

### Sources of Funding

This work of C.M. was supported by the Japanese Society for the Promotion of Science (JSPS) Kakenhi grant number JP19K17813, the Naito Foundation, and the Medical Research Encouragement Prize of The Japan Medical Association.
